# Freeze-Fracture Replica Immunolabelling Reveals Urothelial Plaques in Cultured Urothelial Cells

**DOI:** 10.1371/journal.pone.0038509

**Published:** 2012-06-29

**Authors:** Mateja Erdani Kreft, Horst Robenek

**Affiliations:** 1 Institute of Cell Biology, Faculty of Medicine, University of Ljubljana, Ljubljana, Slovenia; 2 Leibniz-Institute for Arteriosclerosis Research, University of Münster, Münster, Germany; Institute for Virus Research, Laboratory of Infection and Prevention, Japan

## Abstract

The primary function of the urothelium is to provide the tightest and most impermeable barrier in the body, i.e. the blood-urine barrier. Urothelial plaques are formed and inserted into the apical plasma membrane during advanced stages of urothelial cell differentiation. Currently, it is supposed that differentiation with the final formation of urothelial plaques is hindered in cultured urothelial cells. With the aid of the high-resolution imaging technique of freeze-fracture replica immunolabelling, we here provide evidence that urothelial cells *in vitro* form uroplakin-positive urothelial plaques, localized in fusiform-shaped vesicles and apical plasma membranes. With the establishment of such an *in vitro* model of urothelial cells with fully developed urothelial plaques and functional properties equivalent to normal bladder urothelium, new perspectives have emerged which challenge prevailing concepts of apical plasma membrane biogenesis and blood-urine barrier development. This may hopefully provide a timely impulse for many ongoing studies and open up new questions for future research.

## Introduction

Urothelial plaques are ultrastructurally distinctive, highly ordered structures made of crystalline arrays of 16-nm hexagon shaped protein particles consisting of four integral membrane proteins called uroplakins i.e. UPIa, UPIb, UPII and UPIIIa [1], [2]. The latter are the most significant molecules in the apical plasma membrane (PM) of the differentiated urothelial cells (UCs) that line the urinary tract from the renal pelvis to the proximal urethra [3], [4]. Uroplakins contribute to the blood-urine barrier, the tightest and most impermeable barrier in the body, by their structural organization [5] and by hindering endocytosis from the apical PM [6].

Although uroplakins were previously found in cultured UCs [7]–[11], the prevailing view is that differentiation with the final formation of urothelial plaques is hindered in cultured UCs. Moreover, it is believed that cultured UCs revert to a more undifferentiated “primitive” phenotype [7], [12]. The widely held assumption that uroplakins do not form urothelial plaques in cultured UCs is based on immunofluorescence light microscopy and standard thin section electron microscopy (EM).

In view of these conflicting data, we have now sought detailed evidence for or against the presence of urothelial plaques positive for uroplakins in cultured UCs by using the high-resolution imaging technique of freeze-fracture EM combined with immunogold labelling, i.e. freeze-fracture replica immunolabelling (FRIL). Furthermore, the ultrastructural differentiation of cultured UCs was determined by thin section and scanning EM and the barrier function was assessed by measurement of transepithelial electrical resistance (TER).

Using these different experimental approaches, we show that in long-term cultures the UCs form an urothelium, in which the superficial UCs express uroplakins and form uroplakin-positive urothelial plaques indistinguishable from those of superficial UCs *in vivo*. Our findings illustrate the recent impact of the FRIL technique in demonstrating the localization of uroplakins and the structure and formation of urothelial plaques in UCs both *in vivo* and *in vitro*.

## Materials and Methods

### Specimens and Chemically Defined Medium for Urothelial Cells

The experiments were approved by the Veterinary Administration of the Slovenian Ministry of Agriculture and Forestry (permit no. 34401-1/2010/6) in compliance with the Animal Health Protection Act and the Instructions for Granting Permits for Animal Experimentation for Scientific Purposes. Urinary bladders were obtained from adult male mice; strain ICR CD1. Bladders were handled aseptically and immediately immersed in medium for urothelial cells, i.e. UroM consisting of equal parts of MCDB 153 medium (Sigma, Taufkirchen, Germany) and Advanced-DMEM medium (Invitrogen, Gibco, Paisley, UK) and supplemented with adenine (15 µg/ml), insulin (5 µg/ml), hydrocortisone (0.5 µg/ml), phosphoetanolamine (0.1 M), glutamax (4 mM), streptomycine (100 µg/ml) and penicillin (100 µg/ml). The final Ca^2+^ concentration was 2 mM. Normal UCs, gently scraped with a scalpel blade from the bladder of an adult male mouse, were used as a control, i.e. UCs *in vivo*.

### Long Term Primary Mouse Urothelial Culture

Primary mouse urothelial cultures were prepared as described previously [10], [13]. Briefly, the urothelium and underlying lamina propria were separated from the submucosa and muscle layer mechanically using sterile forceps. The isolated mucosa was cut into explants (2–3 mm^2^, 10–12 explants for each mouse bladder), which were transferred onto 0.4 µm porous membranes (BD Falcon, Bedford, USA). Each explant was orientated and spread out, so that the urothelium was on the upper side. Culture medium was introduced into the well containing the porous membrane, so that the urothelium was positioned at the air-liquid interface. Medium was changed daily with the exception of weekends. After 2 months, the molecular and ultrastructural status of UCs growing onto the porous membrane was analyzed.

### Transepithelial Resistance (TER)

TER across the urothelia grown on porous membranes (BD Falcon) was measured for 4 months (from the 4^th^ week, when the UCs reached confluence till the 17^th^ week). Cultured urothelia were grown in medium UroM for 14 weeks. Then one-third of cultured urothelia was propagated in a medium supplemented with 2.5% fetal bovine serum (FBS, Gibco) and two-thirds were propagated in the same medium for an additional 3 weeks. For measuring TER an epithelial voltohmmeter (EVOM & EVOMX, Sarasota) and STX2 electrodes were used. The measured TER was corrected by subtracting the mean resistance of blank porous membranes (150 Ωcm^2^) and the results were expressed as Ωcm^2^ ± SE. Urothelial TER values were averaged and compared by a two-tailed Student's t-test; p values less than 0.05 were considered statistically significant.

### Thin Section and Scanning Electron Microscopy

UCs were prepared for thin section and scanning EM as described previously [9]. In brief, after 2 months of culturing the UCs were fixed in 4% (w/v) paraformaldehyde and 2.5% (v/v) glutaraldehyde in 0.1 M cacodylate buffer, pH 7.4 for 2 h 45 min. The fixation was followed by overnight rinsing in 0.1 M cacodylate buffer. The samples were then postfixed in 1% (w/v) osmium tetroxide for 1 h at 4°C, dehydrated in a graded series of ethanol and embedded in Epon (Serva Electrophoresis, Heidelberg, Germany). Ultrathin sections were contrasted with uranyl acetate and lead citrate and observed in a Philips CM100 transmission electron microscope. After dehydration through a graded series of acetone, the samples for scanning EM were dried at the critical point, spattered with gold and observed in a Jeol 840A scanning electron microscope.

### Freeze-fracture Replica Immunolabeling (FRIL)

**Figure 1:**
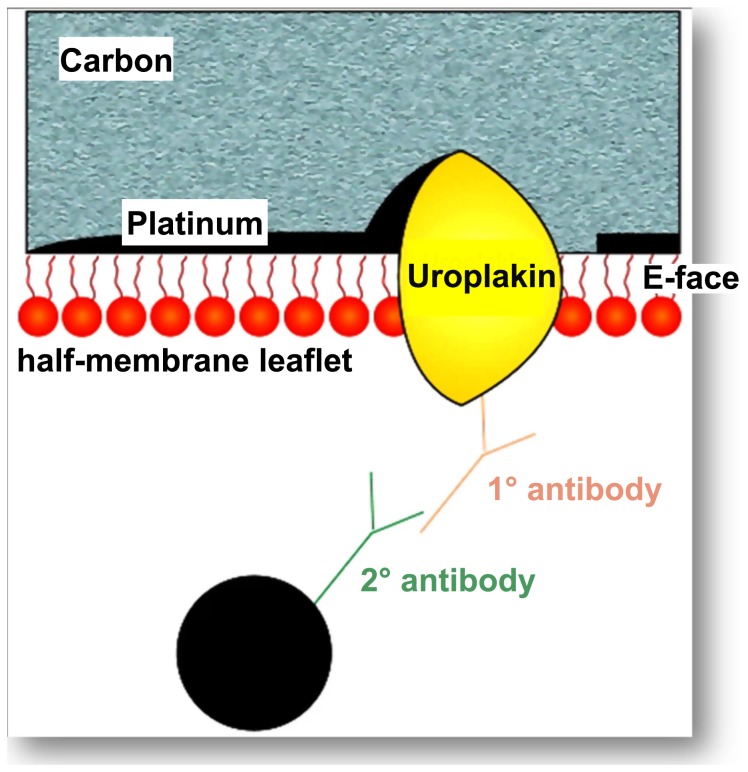
Diagram illustrating the principle of FRIL to visualize the location of uroplakins at high resolution in membranes. The technique permits retention of a layer of molecules attached to the platinum-carbon replica. The term “fracture-face” is reserved for the interior views of membranes exposed by freeze fracturing. The term E face is used for the half-membrane leaflet adjacent to the extracellular space, while the term P face is used for that adjacent to the protoplasm (i.e. cytoplasm). The integral membrane proteins uroplakins embedded in the replica can be labelled using a primary antibody followed by a secondary antibody coupled to colloidal gold. On examination in the transmission electron microscope, the electron dense gold label is clearly visible against the replica, marking the uroplakins in the plane of the membrane.

This technique involves 1. splitting membranes at low temperatures to give extensive planar views of the membrane interior, 2. replicating the membrane’s structural detail by vacuum deposition of platinum and carbon, 3. removing all but a molecular layer of the cells by washing with sodium dodecyl sulphate, and 4. applying antibody labelling techniques with electron-dense gold particles to localize the uroplakin-positive urothelial plaques (Fig. 1).

Full description of the methodology and nomenclature has been reported previously [14], [15]. In brief, UCs were cultured in medium UroM at 37°C, 5% CO_2_ for 2 months. We performed three independent FRIL experiments. Each experiment was performed on the long-term primary urothelial cultures established from the mouse urinary bladder explants. In sum, we analyzed 23 long-term primary urothelial cultures prepared from three urinary bladders. After 2 months, the explants were removed and only the UCs growing on the porous membranes were rapidly frozen, freeze-fractured and platinum-carbon replicas were made following the standard protocol used for freeze-fracture electron microscopy. However, instead of removing the biological material from the replica with bleach or acids, as in the conventional technique, the replica was treated with Tris-bufferd 5 % sodium dodecyl sulphate (SDS) with 30 mM sucrose, pH 8.3, overnight at room temperature. The SDS removed the bulk of the biological material from the replica so that structure was visible by EM, leaving a single lipid monolayer and associated integral and surface proteins adherent to the replica (Fig. 1). This remaining layer was so thin that it did not obstruct the electron beam. After SDS treatment the replicas were thoroughly washed in PBS and incubated in blocking buffer (5% BSA, bovine serum albumin). Uroplakins were then localized by immunogold labelling. Primary rabbit polyclonal antibodies against all four uroplakins were diluted 1∶5000. The antibodies used were a rabbit polyclonal anti-asymmetric unit membrane (AUM) antibody (a gift from Professor Dr. T-T Sun), which is generated against total mature uroplakins [16]. Secondary antibodies the goat anti-rabbit 18-nm gold complexes were from Jackson Immunoresearch, West Grove, NA, USA. Immunolabelled replicas were observed in a Philips 410 transmission electron microscope.

**Figure 2:**
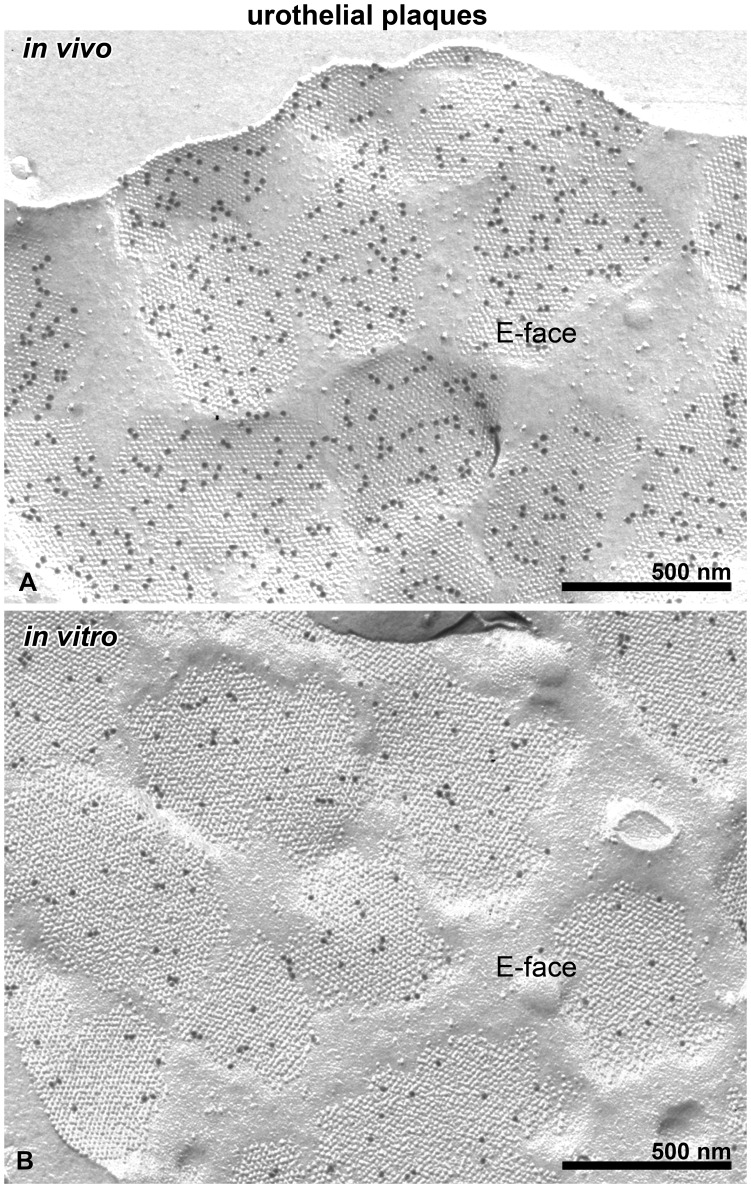
Urothelial plaques are detected in the apical PM of UCs *in vivo* and *in vitro*. Immunogold labelling for uroplakins is seen on the E faces of the apical PM of UCs *in vivo* (A) and *in vitro* (B). The maximum calliper diameters and the morphology of urothelial plaques are similar in UCs *in vivo* and *in vitro*.

## Results and Discussion

In order to determine whether the uroplakins particles are formed in UCs *in vitro* and to examine the possibilities of their arrangement into the urothelial plaques, we generated a long-term primary mouse urothelial culture. Using different experimental approaches, we determined the molecular and ultrastructural differentiation and the functional property of *in vitro* developed urothelium. We applied FRIL, which here provides unequivocal evidence that UCs *in vitro* form uroplakin-positive urothelial plaques.

**Figure 3:**
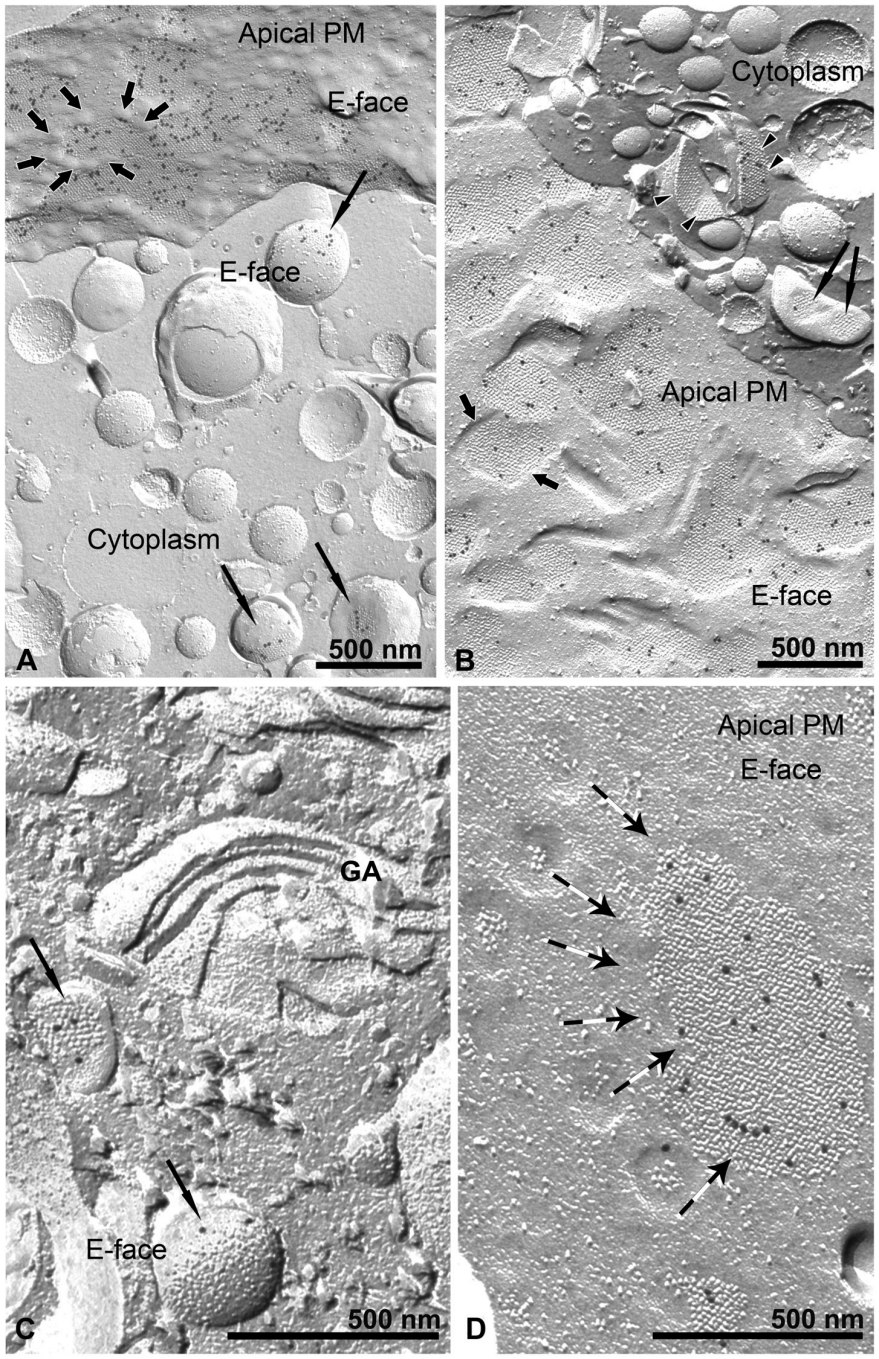
Urothelial plaque biogenesis in DFVs and apical PM of UCs *in vitro*. Images of urothelial plaques in the apical PM and membranes of DFVs of UCs *in vitro* prepared by freeze-fracture electron microscopy with immunogold labelling for uroplakins. Abundant immunogold label is seen in urothelial plaques on the E faces of the apical PM (A, B, short arrows), and also on E faces of DFV membranes (A, B, C, long arrows, and in B, arrowheads). DFVs with uroplakin label could be seen near the Golgi apparatus (GA) (C, long arrows), from which probably the DFV membranes are derived, and in close proximity to the apical PM (A, B). The number and ordering of uroplakin particles in urothelial plaques varies between DFVs. The DFVs in B, marked with arrowheads, bear more uroplakin particles than those marked with long arrows. This could be interpreted as the sequential assembly of uroplakin particles into DFV membranes. Variations in the number of uroplakin particles in the urothelial plaques are also seen in the apical PM (A, B), suggesting that the gradual aggregation of small urothelial plaques into larger ones is not only limited to DFVs but still takes place in the apical PM (D, long arrows). The edges of urothelial plaques appear rounded (A, thick arrows) or straight (B, thick arrows).

**Figure 4:**
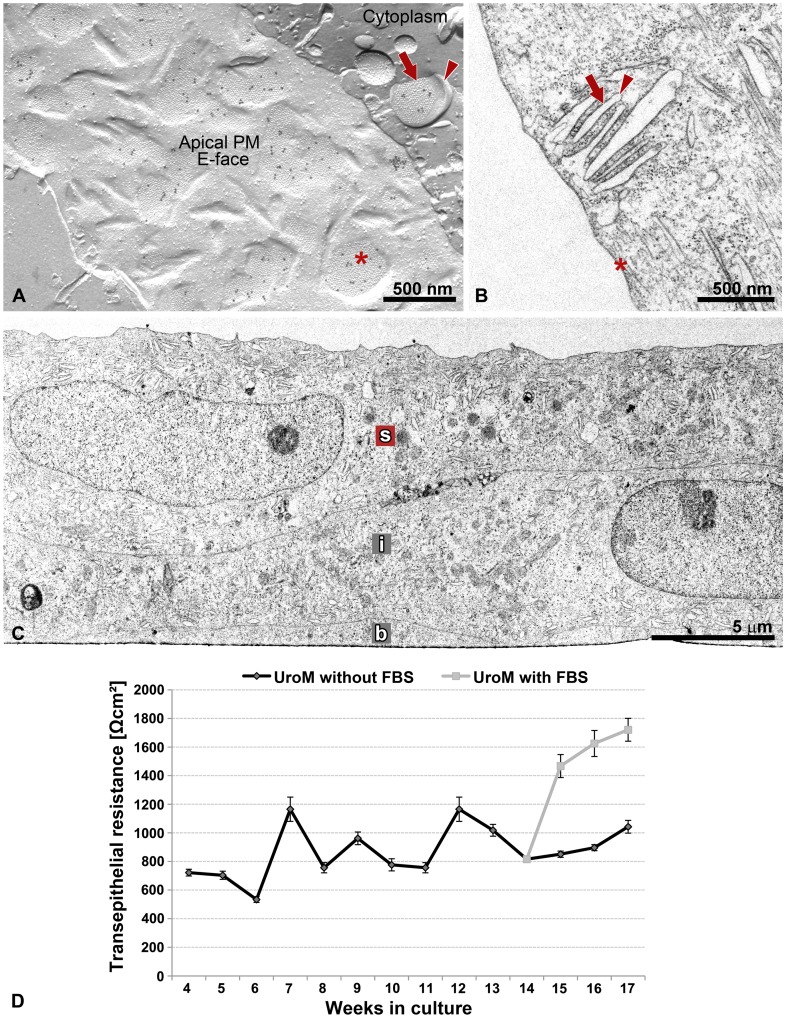
Urothelium *in vitro* with the ultrastructural and functional properties of native urothelium. (A) FRIL, (B, C) thin section EM, (D) TER measurements. (A) Image of urothelial plaques in the apical PM and membrane of DFV of UC *in vitro* prepared by freeze-fracture electron microscopy with immunogold labelling for uroplakins. The uroplakin-positive urothelial plaques in the apical PM (asterisk) and the cytoplasm (arrow) correspond to (B) rigid-looking, concave shaped apical PM structures (asterisk in B) and membranes of the DFVs (arrow in B), respectively, that are visible by thin section EM. Hinge regions are marked by arrowheads in A and B. Note close association of DFVs with the apical PM in A and B. (C) The UCs *in vitro,* like UCs *in vivo,* are organized into the urothelium with the basal (b), intermediate (i) and superficial (s) cells. (D) The TER of urothelia grown on porous membrane was measured for 4 months, from week 4 onwards. Each point represents the mean TER (±SE) of urothelia. Urothelia were grown for 14 weeks in the medium UroM without fetal bovine serum (FBS, n = 12), then the two-thirds of urothelia were propagated in the same medium (n = 8) and the other one-third in UroM with 2.5% FBS (n = 4) for an additional 3 weeks. Note the significant increase in the TER of urothelia, which were transferred from the medium UroM without FBS to UroM with FBS.

**Figure 5:**
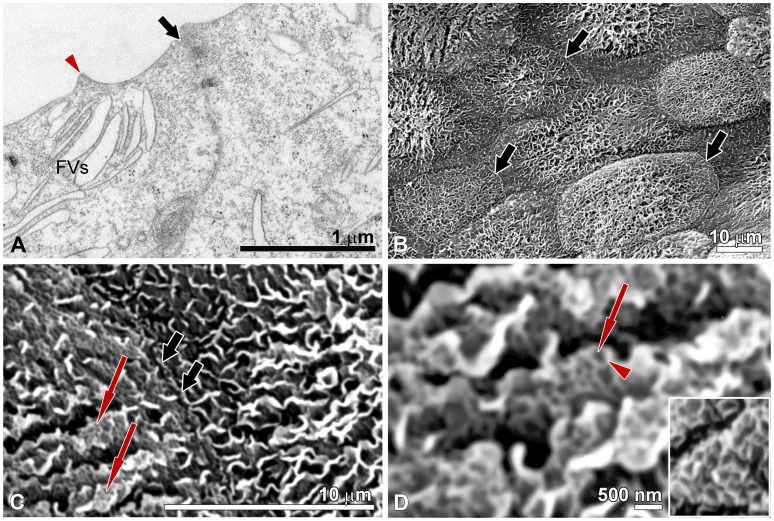
Urothelium *in vitro* with the tight junctions and scalloped appearance of the apical PM. (A) Thin section EM and (B–D) Scanning EM. (A) Superficial UCs are connected with well-developed cell junctions (noticeable in the direction of thick arrow). Note accumulation of FVs close to the apical PM. The hinge region is marked with arrowhead. (B) Overview of the apical surface showing the superficially-positioned UCs and well developed cell borders between them (thick arrows). (C) The surface topography of superficial UCs *in vitro* reveals the scalloped appearance of the apical PM. Arrows denote the regions, which in the FRIL and thin section EM micrographs correspond to the urothelial plaques. Tight junction is seen between two superficial UCs (thick arrows). (D) At higher magnification the scanning EM shows the urothelial plaques as darker areas of hexagonal morphology (arrow) and hinge regions as brighter ridges (arrowhead). In inset, at the same magnification the apical PM with urothelial plaques and the hinge regions in superficial UC *in vivo*.

### Urothelial Cells *in vitro* Form Uroplakin-positive Urothelial Plaques

Figure 2 shows the localization of uroplakin-positive urothelial plaques in normal mouse UCs *in vivo* (Fig. 2A) and in UCs propagated *in vitro* for 2 months (Fig. 2B). The apical PM of UCs *in vitro* exhibits plaques indistinguishable from those of UCs *in vivo*. The plaques were found in all cultures analyzed. There was no significant difference (P>0.05) between the maximum calliper diameter of urothelial plaques in UCs *in vivo* (744±43 nm, n = 30) and *in vitro* (710±50 nm, n = 30). Variations in the number of uroplakin particles in the urothelial plaques were found in both UCs *in vivo* and *in vitro*. The immunogold label was confined to the E face of the apical PM (Fig. 2); no label was detected on the P face in accordance with previous studies demonstrating the (uroplakin) subunits to be located in the external half-membrane leaflet, penetrating through to the exterior [17]. The terms E face and P face are applied to the interior view of the half-membrane leaflets of the PM that lie adjacent to the extracellular space and the protoplasm, i.e. cytoplasm, respectively (Fig. 1).

Apart from positive labelling of uroplakins on the apical PM E face, prominent labelling was apparent on the E faces of discoidal- or fusiform-shaped vesicles (DFVs) in the cytoplasm (Fig. 3A, B). DFVs are involved in the transport of uroplakins to the apical PM [18]–[21]. Freeze-fracture images disclose that DFVs are often seen in close association with the apical PM (Fig. 3A, B) and the Golgi apparatus (Fig. 3C). This is in accordance with our previous observations [19] and earlier studies on intact tissue [20]. The Golgi apparatus did not contain uroplakin-positive urothelial plaques, which is in agreement with the study of Hudoklin et al [21], which show no uroplakin labelling of the Golgi apparatus on cryo-ultrathin sections. The size of urothelial plaques on the membrane of DFVs resemble those found in close proximity to larger ones in the apical PM (Fig. 3D). The obvious associations of DFVs containing urothelial plaques in the cytoplasm and the apical PM revealed in this study indicate that these features play important roles in urothelial plaque biogenesis and growth. These associations are ideally configured to function in the intracellular synthesis and transport as well as the cytoplasmic-plasmalemmal transfer and the progressive incorporation of uroplakins into urothelial plaques in the apical PM.

### In long-term Cultures the UCs Develop Ultrastructural and Functional Characteristics of Highly Differentiated Superficial UCs *in vivo*

Urothelial plaques are unique and specific assemblies of highly differentiated superficial UCs composed of uroplakins [3]. In this study, we demonstrated that by using FRIL we can unequivocally show that rigid-looking, concave shaped membrane structures that are visible by thin section EM (asterisk in Fig. 4B) and the darker areas of hexagonal morphology that are visible by scanning EM (arrows in Fig. 5C, D) correspond to uroplakin-positive urothelial plaques (asterisk in Fig. 4A). Additionally, we proved that the cytoplasmic DFVs containing uroplakin-positive urothelial plaques (arrow in Fig. 4A) and narrow rims of membranes without particles – termed hinges (arrowhead in Fig. 4A) correspond to DFVs that are visible by thin section EM (arrow and arrowhead in Fig. 4B). These results further support the idea that UCs *in vitro* retain the capability for synthesis of the uroplakin particles and their arrangement into urothelial plaques.

In order to examine whether our *in vitro* established urothelia (Fig. 4C) are “tight” epithelia with a TER >500 Ωcm^2^, according to the definition of Fromter and Diamond [22], we measured the TER of cultured UCs growing on a porous membrane for 4 months (Fig. 4D). We started to measure TER in the 4^th^ week, when the confluence was reached in each of the UC cultures (n = 12). TER measurements over the course of the 4 months revealed that urothelia grown in such culture conditions form “tight” epithelia. Urothelia grown in a medium UroM without FBS had a mean TER from 534 ± 20 Ωcm^2^ to 1165 ± 85 Ωcm^2^.

Thin section and scanning EM of the long-term urothelial cultures clearly demonstrated well-developed tight junctions between superficial UCs (Fig. 5A–C). Our previous immunocytochemical studies on primary urothelial cultures have already revealed occludin-, ZO-1-, claudin-4- and claudin-8-containing tight junctions between the new superficial UCs in 7-day primary urothelial cultures [13]. We therefore suggest that in our *in vitro* established long-term normoplastic urothelial cultures, the high TER is maintained by the well-developed cell junctions, and not with the uroplakin-positive urothelial plaques. This interpretation is consistent with the findings of Hu and co-workers [5], which demonstrated that UPIII-deficient urothelium exhibits a normal TER but has significantly elevated water permeability and to a lesser extent also increased urea permeability. Additional studies are underway to better define the permeability parameters of the long-term urothelial cultures.

To evaluate the viability and functional responsiveness of the long-term urothelial culture, one-third of cultured urothelia was transferred into a medium supplemented with 2.5% FBS in the 14^th^ week. TER measurements revealed that addition of 2.5 % FBS induces the significant increase in TER (Fig. 4D). From the 14^th^ to 17^th^ week the urothelia grown with FBS had enhanced the barrier function. TER measurements over the course of the 3 weeks revealed a mean TER from 816 ± 22 Ωcm^2^ to 1721 ± 45 Ωcm^2^.

These findings show the significant effect of serum on urothelial TER, which is in agreement with our recent study on the hyperplastic urothelial models [23]. They further confirmed the functional capacity of the urothelial *in vitro* model. Moreover, the findings support the idea that UCs in long-term urothelial culture retain their viability and still respond to extracellular signals, e.g. the added serum.

### Conclusions

Our results have a number of applications. The established urothelial culture model can be used as a research tool for investigating the cellular-molecular mechanisms of apical PM biogenesis, which are crucial for development of the blood-urine barrier. The prevailing hypothesis proposes that differentiation with the final formation of urothelial plaques is hindered in cultured UCs. By revisiting the localization of uroplakins in cultured UCs with the FRIL technique, the first unequivocal evidence for uroplakin-positive urothelial plaques in UCs *in vitro* has been achieved. Apart from positive labelling of uroplakins in cytoplasmic DFVs and apical PM, FRIL has also provided new insights into the dynamics of urothelial plaque formation. Among the key questions to be addressed in future studies are whether additional types of protein other than uroplakins are involved in urothelial plaque formation and how the final size of an urothelial plaque is determined and regulated.

To sum up, the findings discussed illustrate the recent impact of the FRIL technique in demonstrating the localization of uroplakins and the structure and formation of urothelial plaques in UCs both *in vivo* and *in vitro*. The information this approach provides is unique and we expect that further exploitation of the FRIL technique in future studies may advance our understanding of the biology of the blood-urine barrier.
